# Protective Effects of Alkaloid Compounds from *Nelumbinis Plumula* on *tert*-Butyl Hydroperoxide-Induced Oxidative Stress

**DOI:** 10.3390/molecules180910285

**Published:** 2013-08-26

**Authors:** Yong Xie, Yi Zhang, Long-Tao Zhang, Shao-Xiao Zeng, Ze-Bin Guo, Bao-Dong Zheng

**Affiliations:** 1 College of Food Science and technology, Fujian Agriculture and Forestry University, Fuzhou 350002, Fujian, China; 2 College of Pharmacy, Fujian University of Traditional Chinese Medicine, Fuzhou 350122, Fujian, China

**Keywords:** cytoprotection, alkaloid, oxidative stress, *Nelumbinis Plumula*

## Abstract

This study was conducted to investigate the effect of *Nelumbinis Plumula* total alkaloid (NPA) and its main alkaloid components on oxidative stress induced by *tert*-butyl hydroperoxide (*t*-BHP) in the human hepatocellular HepG2 cell line. According to HPLC analysis, several major alkaloid compounds such as liensinine, isoliensinine and neferine were present in NPA. The cytotoxic effects in 0.55 mM *t*-BHP-induced HepG2 cells were significantly inhibited by NPA and the major compound in NPA, neferine, showed the strongest activities. The protective effect of neferine against oxidative stress induced by *t*-BHP may be associated with decreased ROS formation, TBARS generation, LDH release and increased GSH levels, suggesting their involvement of the cytoprotective on oxidative stress. The effects were comparable with quercetin, which was used as positive control. Overall, total alkaloid and alkaloid compounds from *Nelumbinis Plumula* displayed a significant cytoprotective effect against oxidative stress. Further study is needed to elucidate the relationship between the chemical structures of the components in NPA and their protective effect on oxidative stress.

## 1. Introduction

Clinical and experimental studies suggest that excessive reactive oxygen species (ROS) may bring about oxidative stress and thus attack cellular biomolecules such as lipids, proteins and DNA [1]. The oxidative stress-induced damage can disrupt cellular function and membrane integrity, thereby leading to cell death [2]. Moreover, since hepatocytes make up the majority of liver structures and they are susceptible to toxicants, oxidative stress of hepatocytes caused by toxicants may be associated with liver diseases such as hepatotoxicity [3].

Natural antioxidants may play an important role in lengthening the shelf life of cellular biomolecules such as DNA, proteins and lipids, reducing cancer or death of cells and harmful substances by regulating oxidative stress [4]. Thus, a main protective strategy against oxidative damage of hepatocytes that are susceptible to cell injury is through the induction of antioxidants in cells by substances that show cytoprotective effects.

Lotus seeds have been widely consumed by people in Asia. Different parts of the lotus such as germs, leaves and rhizomes have been reported for their chemical compounds and biological activities. Hu *et al*. [5] reported the *in vivo* and *in vitro* antioxidant activity of phenolic compounds, carotene, alkaloids and saponins from germs, leaves or rhizomes of the lotus. The therapeutic hepatoprotective activity of ethanolic extracts of edible lotus leaves was also studied, indicating that the hepatoprotective activity of lotus leaf extract (LLE) was comparable with that of a standard treatment comprising silymarin, a known hepatoprotective drug [6]. *Nelumbinis Plumula* is the germ of the lotus seeds, which tastes bitter and is generally removed. Several bioactive sections have been derived from parts of *Nelumbinis Plumula*, including alkaloids, flavonoids, triterpenoids, polysaccharides, *etc*., which have been reported to possess functional activities [7]. There has been considerable interest in components from *Nelumbinis Plumula* oil, such as sterols, vitamins, unsaturated fatty acids, and phenol compounds that were analyzed by gas chromatography-mass spectrometry (GC-MS) [8]. The hepatoprotective activities of the oil were studied by examining its effect on carbon tetrachloride-induced chronic hepatotoxicity in mice, which may be recognized as a powerful functional activity against oxidative stress [9]. Apart from lipid-soluble constituents, water extracts from *Nelumbinis Plumula* may also show antioxidant activities. *In vitro* research indicated that water extracts from *Nelumbinis Plumula* can scavenge oxygen radicals and inhibit lipid peroxidation [10]. Further proof may be acquired by *in vivo* tests, showing that polysaccharide extracted from the plant may have potential to treat type 1 diabetes via its potent anti-inflammatory and antioxidant activities [11]. Moreover, the major component, liensinine, was reported to produce a significant increase of GSH-Px and SOD activities in experimental hyperlipidemia rats, showing relative high inhibition of lipid peroxidation [12].

The study of the effect of antioxidants on the regulation of defenses against liver injuries may benefit from the use of an established cell culture line, human hepotoma HepG2. HepG2 cells are considered a good model to study *in vitro* metabolism and toxicity to the liver, since they retain many of the specialized functions which characterize normal human hepatocytes [13]. Challenged by *tert*-butyl hydroperoxide, HepG2 can be used to establish a reliable model for evaluating the protective effects against oxidative stress of different kinds of antioxidants [14].

In our previous studies, we have compared different parts of lotus *Nelumbinis Plumula* for their antioxidant activities *in vitro* and concluded that the total alkaloid part possessed the strongest activities (data not reported). The objective of the present study was to investigate the hepatoprotective effect of *Nelumbinis Plumula* alkaloid against oxidative stress induced in HepG2 cells by *tert*-butyl hydroperoxide.

## 2. Results and Discussion

### 2.1. Total Alkaloid Content and DPPH Radical-Scavenging Activities of Nelumbinis Plumula Total Alkaloid (NPA)

The total alkaloid content (TAC) of NPA was determined by an acid dye complexing colorimetric method. TAC in NPA was thus measured as 52,059 mg liensinine perchlorate equivalents/100 g dry weight. The antioxidant activity of NPA was evaluated by a DPPH radical-scavenging assay. DPPH is a quite stable free radical that shows a maximum absorption at 516 nm, which may be reduced by adding some antioxidant into the system [15], so we can determine the antioxidant activity potency by measuring the DPPH radical-scavenging activity. In the present study, it was observed that the DPPH free radical scavenging activity was concentration dependent in NPA and reached a maximum at a concentration of 200 μg/mL, which showed the highest inhibition rate of 76.8% (Table 1). The 50% effective concentration of scavenging activity in NPA, which was expressed as an EC_50_ value, can reach as high as 82.56 μg/mL. Although the EC_50_ dose of NPA may be regarded as supraphysiological since our previous experiments showed that NPA at concentrations above 50 μg/mL may already show some cytotoxic effects in HepG2 cells, the results for the *in vitro* DPPH radical-scavenging assay also indicate that NPA exhibited definite antioxidative properties.

**Table 1:** Results of DPPH radical-scavenging activity for NPA with different concentration. Data are expressed as the means ± standard deviation (n = 3). Values with different upper letters in different treated groups indicate a significant difference (*p* < 0.05).

	NPA concentration (μg/mL)					Control
	12.5	25	50	100	200	Ascorbic acid *
**Percentage of inhibition (%)**	11.95 ± 0.7 ^a^	16.62 ± 1.5 ^a^	54.46 ± 4.9 ^b^	67.19 ± 5.7 ^c^	76.8 ± 4.6 ^d^	88.52 ± 7.8 ^e^

* 40 μg/mL.

### 2.2. UV-Vis Spectrophotometric Analysis and HPLC Analysis of NPA

**Figure 1:**
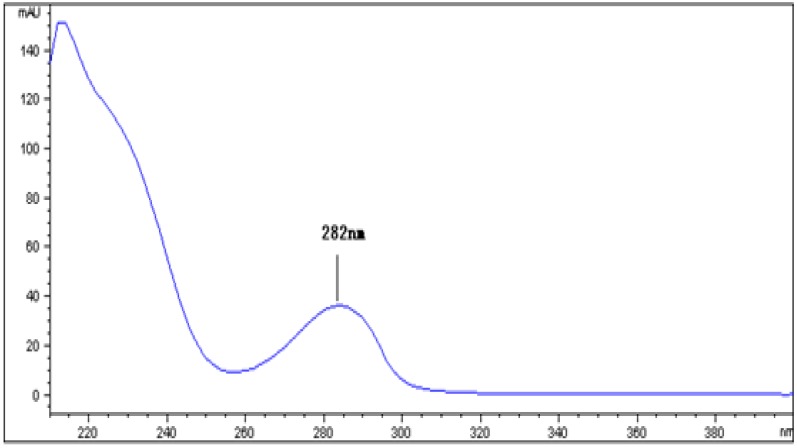
UV-vis spectra of *Nelumbinis Plumula* total alkaloid (NPA). Peak 1 was the characteristic absorption peak detected at the wavelength of 282 nm.

**Figure 2:**
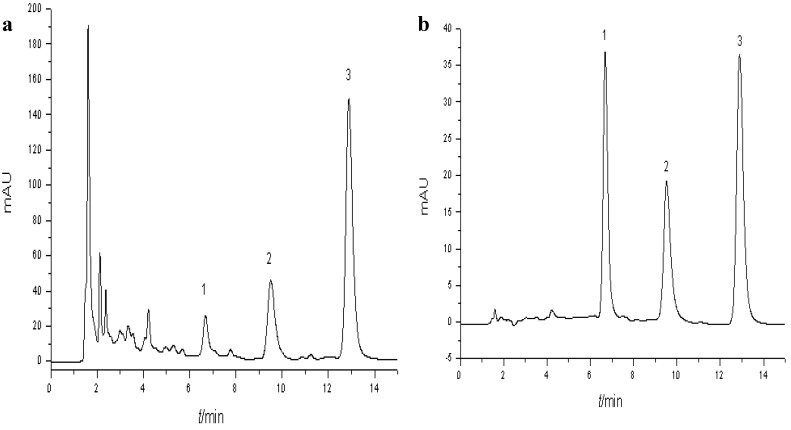
(**a**) Analytical HPLC chromatogram of *Nelumbinis Plumula* alkaloid (NPA) (**a**) and the three alkaloid standard compounds. (**b**) Peaks 1–3 correspond to the compounds liensinine (peak 1), isoliensinine (peak 2) and neferine (peak 3), respectively.

**Figure 3:**
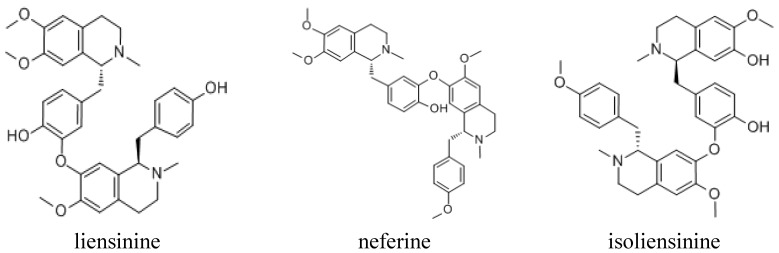
The structure of the three alkaloid compounds, liensinine, isoliensinine and neferine.

As shown above, NPA showed significant *in vitro* DPPH radical-scavenging activity. The major compounds of NPA were identified by HPLC using in-line diode array detector (DAD) monitoring at 282 nm of the UV-Vis absorption. As shown in Figure 1, the characteristic absorption peak of NPA was detected at 282 nm. The HPLC chromatogram of NPA was complicated, indicating three major peaks showing identical retention times with standards of the alkaloids liensinine, isoliensinine and neferine, respectively (Figure 2 and Figure 3), which make up the major components in *Nelumbinis Plumula* alkaloids [16]. Based on the comparison of peak areas of each peak with those of authentic samples, liensinine, isoliensinine and neferine were quantified to be about 2.57%, 4.38% and 9.52%, respectively.

### 2.3. Protective Effect of NPA and its Alkaloid Compounds on Cytotoxicity in *t*-BHP-Induced HepG2 Cells

**Figure 4:**
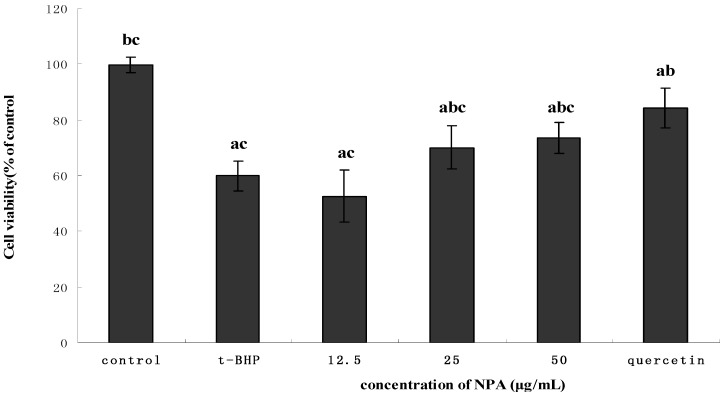
Effect of different concentrations of NPA on *t*-BHP-induced cytotoxicity in HepG2 cells. The cells were pretreated with NPA for 24 h, then the alkaolid conditioned media was removed and replaced by 0.55 mM *t*-BHP. After incubating for 4 h, the medium was removed and cell viability was determined by the MTT method. The cell viability of control cells was defined as 100%. Data are presented by means ± SD (n = 3). ^a^ indicates a significant difference from the control group (*p* < 0.05); ^b^ indicates a significant difference from the *t*-BHP-treated group (*p* < 0.05); ^c^ indicates a significant difference from the quercetin-treated group (*p* < 0.05)

*t*-BHP has been widely used to study the effects of antioxidants in phytochemicals from natural products [17,18]. In the present study, the treatment of the HepG2 cell line with *t*-BHP, at a concentration of 0.55 mM, can produce a significant decrease in cell viability of up to 53.2% compared with the control groupas revealed by the cytotoxicity assay (*p* < 0.05, see Figure 4).

To evaluate whether NPA or its components can protect HepG2 cells from oxidative stress induced by 0.55 mM *t*-BHP and thus increase cell viability, we first determined the effects of NAP at concentrations in the 0–50 μg/mL range or individual alkaloid compounds (liensinine, isoliensinine and neferine) at concentrations ranging from 0–40 μM on the growth of HepG2 cells affected by *t*-BHP. The results showed that none of the samples above showed any cytotoxic effects in HepG2 cells after 24 h of treatment (data not shown). Thus, these concentration ranges were used for further experiments. As shown in Figure 4, 0.55 mM *t*-BHP can significantly decrease the viability of cells, while treatment of HepG2 cells with NPA at concentration of 25 or 50 μg/mL can significantly protect cells against *t*-BHP-induced cytotoxicity (*p* < 0.05). As for quercetin used as positive control, exposure of HepG2 cells to quercetin for 24 h at concentrations as high as 83 μM, which has not shown any signs of cytotoxocity [19], can also significantly increase cell viability of *t*-BHP-treated HepG2 cells compared with the NPA-treated or control group (*p* < 0.05).

**Figure 5:**
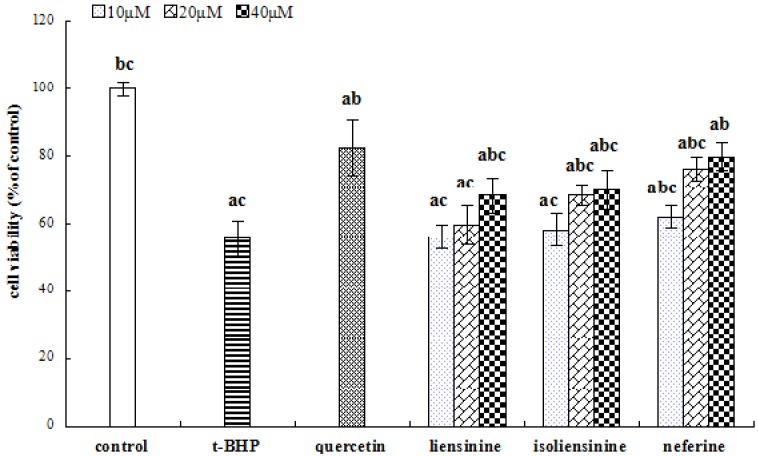
Effects of different concentrations of three alkaloid compounds (liensinine, isoliensinine and neferine) on cytotoxicity of *t*-BHP-treated HepG2 cells. The cells were pretreated with different compounds for 24 h, then the alkaolid conditioned media was removed and replaced by 0.55 mM *t*-BHP. After incubating for 4 h, the medium was removed and cell viability was determined by the MTT method. The cell viability of control cells was defined as 100%. Data are presented by means ± SD (n = 3). ^a^ indicates a significant difference from the control group (*p* < 0.05); ^b^ indicates a significant difference from the *t*-BHP-treated group (*p* < 0.05); ^c^ indicates a significant difference from the quercetin-treated group (*p* < 0.05).

As mentioned above, it is proposed that the three main peaks of NPA were the alkaloid compounds liensinine, isoliensinine and neferine, respectively, so we tested the protective effects of these three alkaloid standards on *t*-BHP-induced cytotoxicity in HepG2 cells. The results are shown in Figure 5, where all the alkaloid standards at higher concentration showed a significant improvement in cell viability as compared with the *t*-BHP alone treated group (*p* < 0.05). Among them, cells treated with the compound neferine at a concentration of 40 μM showed the highest cell viability, which was comparable with the positive control of quercetin (*p* > 0.05). Therefore, we used the alkaloid compound neferine as a standard for the study of the protective effects on *t*-BHP-induced oxidative stress.

### 2.4. Effect of Neferine on LDH Leakage of HepG2

**Figure 6:**
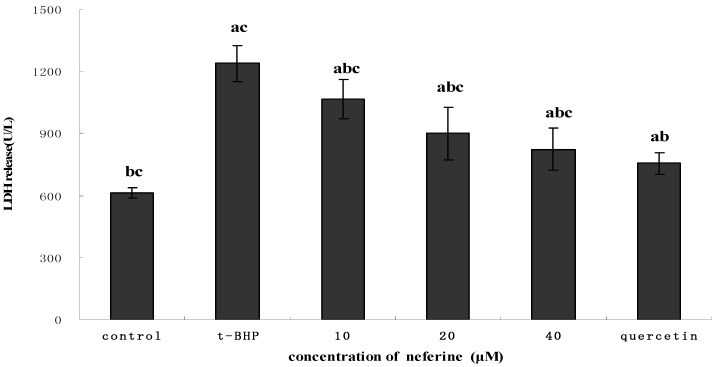
Effect of different concentrations of the compound neferine on LDH leakage in 0.55 mM *t*-BHP-induced HepG2 cells. The cells were pretreated with neferine for 24 h, then the alkaolid conditioned media was removed and replaced by 0.55 mM *t*-BHP. After incubating for 4 h, the medium was collected and determined for LDH content. ^a^ indicates a significant difference from the control group (*p* < 0.05); ^b^ indicates a significant difference from the *t*-BHP-treated group (*p* < 0.05); ^c^ indicates a significant difference from the quercetin-treated group (*p* < 0.05).

In the case of *t*-BHP-induced LDH release, the compound neferine showed significant protective activity (Figure 6). LDH is widely used as a marker to study the toxicity of toxicants on cell death. Numerous studies noted that *t*-BHP can induce an array of cellular dysfunctions, including generation of peroxyl radicals, peroxidation of membrane lipids, and eventually leading to cell death [20]. Our results showed that HepG2 cells treated with 0.55 mM *t*-BHP showed significantly higher LDH release compared with the control group (*p* < 0.05), indicating an unequivocal cell damage in HepG2 (Figure 6). However, the compound neferine can prevent *t*-BHP-induced cell death, as evidenced by the decrease in LDH release from cells after exposure to different concentrations of the compound, indicating the protective effect of HepG2 against cell toxicants.

### 2.5. Protective Effect of Neferine on ROS Generation

In living organisms, *tert*-butyl hydroperoxide treatment is involved in the metabolism of hepatotoxicity, leading to the production of reactive oxygen species (ROS) that initiate lipid peroxidation [21]. Excess ROS can react with many biomolecules such as DNA, lipids [22], and proteins [23], inducing oxidative damage or cell injury in different *in vitro* and *in vivo* systems [24]. To understand whether the observed cytoprotective effect of neferine compound is attributed to the reduction of oxidative stress, we next determined the effect of the sample on intracellular ROS generation of HepG2 cells exposed to *t*-BHP.

**Figure 7:**
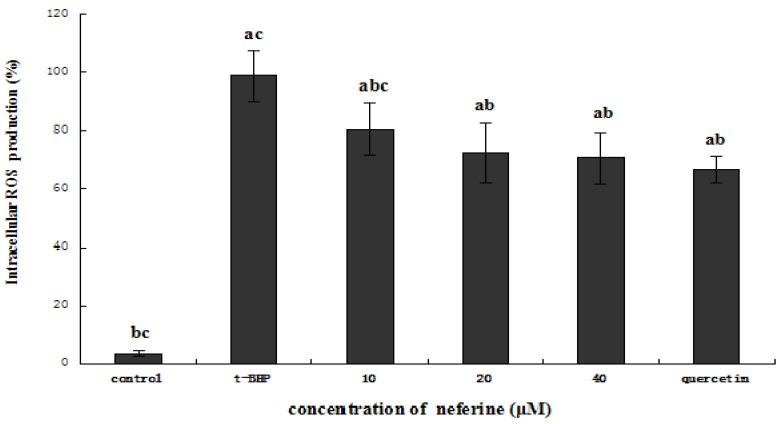
Effect of different concentration of neferine on reactive oxygen species (ROS) in 0.55 mM *t*-BHP-treated HepG2 cells. The cells were pretreated with neferine for 24 h, then the alkaolid conditioned media was removed and replaced by 0.55 mM *t*-BHP. After incubating for 4 h, the medium was removed and lysis buffer was added to each well. The plates were left on ice for 30 min and cell lysates were collected and centrifuged. The supernatants were collected for detection of intracellular ROS. ^a^ indicates a significant difference from the control group (*p* < 0.05); ^b^ indicates a significant difference from the *t*-BHP-treated group (*p* < 0.05); ^c^ indicates a significant difference from the quercetin-treated group (*p* < 0.05).

As shown in Figure 7, compared with the control group, ROS generation increased significantly when the cells were treated with 0.55 mM *t*-BHP (*p* < 0.05), which employs a microsomal cytochrome P-450 system leading to the production of ROS such as alkoxyl radicals [25]. However, when the cells were pretreated with neferine at higher concentrations of 20 or 40 μM, ROS generation was significantly decreased as well as in cells pretreated with quercetin (*p* < 0.05). Thus, it seems that the protective effect of neferine on the cytotoxicity of HepG2 cells induced by *t*-BHP may be partly due to the scavenging of ROS.

### 2.6. Protective Effect of Neferine on Thiobarbituric Acid-Reactive Substance (TBARS)

In order to further evaluate the possible mechanism involved in the protection effects of neferine on cytotoxicity, MDA formation in *t*-BHP-induced HepG2 ells was determined. As discussed above, exposure of HepG2 cells to *t*-BHP can significantly increase intracellular ROS production, which in turn react with the double bonds of polyunsaturated fatty acids (PUFAs) to yield lipid peroxides in the cells [26].

Accumulation of lipid peroxides may alter membrane fluidity and permeability, leading to cytotoxicity of the cells due to the membrane disruption [27]. MDA, a three-carbon compound formed by scission of peroxidized PUFAs, mainly arachidonic acid, is one of the main products of lipid peroxidation [28].

**Figure 8:**
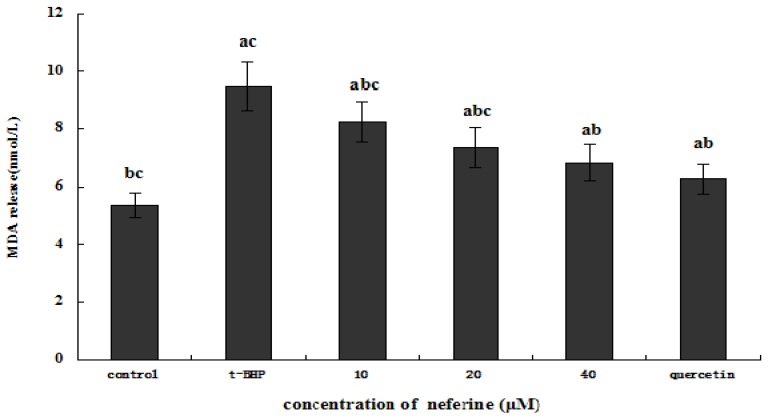
Effect of different concentration of neferine on intracellular concentration of malondialdehyde (MDA) in 0.55 mM *t*-BHP-induced HepG2 cells. The cells were pretreated with neferine for 24 h, then the alkaolid conditioned media was removed and replaced by 0.55 mM *t*-BHP. After incubating for 4 h, the medium was collected and determined for MDA content. ^a^ indicates a significant difference from the control group (*p* < 0.05); ^b^ indicates a significant difference from the *t*-BHP-treated group (*p* < 0.05); ^c^ indicates a significant difference from the quercetin-treated group (*p* < 0.05).

As shown in Figure 8, pretreatment of *t*-BHP-induced HepG2 cells with different concentrations of neferine can significantly reduce the amount of MDA (*p* < 0.05), as compared with *t*-BHP-induced HepG2 cells without neferine treatment. The inhibition effect can reach maximum at the higher concentration of neferine, and showed no significant difference with the positive control group of quercetin (*p* > 0.05). Thus neferine exhibits a remarkable protective activity against the oxidative stress cytotoxicity induced by *t*-BHP by decreasing the degree of lipid peroxidation in the cell.

### 2.7. Effect of Neferine on Intracellular GSH Production in HepG2

Glutathione is a tripeptide, c-l-glutamyl-l-cysteinylglycine, found in all mammalian tissues and in especially high concentration in the liver. Glutathione exists in the thiol-reduced (GSH) and disulfide-oxidized (GSSG) forms [29]. It is well established that GSH is the most important biomolecule protecting against chemically induced cytotoxicity [30], and cellular oxidative stress is often preceded by depletion of intracellular GSH [31]. A major function of GSH is detoxification of toxic oxygen radicals, such as H_2_O_2_ and O_2_^−^, alleviating lipid peroxidation and cell injury. In the process, GSH is oxidized to GSSG, which in turn is reduced back to GSH by GSSG reductase at the expense of NADPH, forming a redox cycle [32].

**Figure 9:**
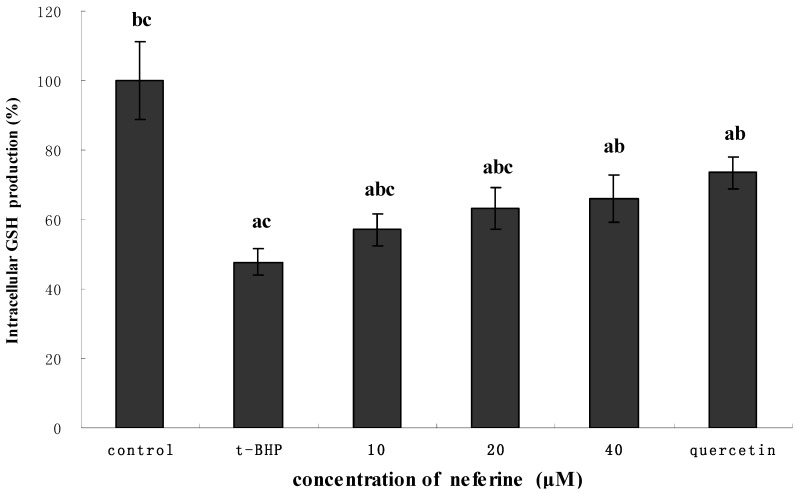
Effect of different concentrations of neferine on intracellular concentration of GSH in 0.55 mM *t*-BHP-treated HepG2 cells. The cells were pretreated with neferine for 24 h, then the alkaolid conditioned media was removed and replaced by 0.55 mM *t*-BHP. After incubating for 4 h, the medium was removed and lysis buffer was added to each well. The plates were left on ice for 30 min and cell lysates were collected and centrifuged. The supernatants were collected for detection of intracellular GSH. ^a^ indicate a significant difference from the control group (*p* < 0.05); ^b^ indicates a significant difference from the *t*-BHP- treated group (*p* < 0.05); ^c^ indicates a significant difference from the quercetin-treated group (*p* < 0.05).

In Figure 9, as compared with the control group, the group treated with *t*-BHP markedly decreased the content of GSH (*p* < 0.05), which was reversed by administration of neferine. The results indicated that neferine can significantly improve the oxidative stress induced by *t*-BHP. It is usually assumed that GSH depletion reflects intracellular oxidation whereas an increase in GSH concentration could be expected to protect the cell against a potential oxidative insult [33]. Reduced glutathione can protect the cells against oxidative damage by providing reducing equivalents to the enzymes of antioxidant system of the cell and by scavenging hydroxyl radicals and singlet oxygen [20]. Hence, maintenance of this tripeptide levels by neferine in this study adds to its cytoprotective role and antioxidant properties.

## 3. Experimental

### 3.1. Reagents

*Nelumbinis Plumula* was purchased from Fujian Xin Zi Jin Curative Co. Ltd. (Fuzhou, Fujian, China). *Nelumbinis Plumula* alkaloid standards (purify > 95%), liensinine, isoliensinine and neferine, 2-Diphenyl-1-picryhydrazyl (DPPH) (purity 99%), *tert*-butylhydroperoxide (t-BHP), ascorbic acid and quercetin was purchased from Sigma Chemicals Co. (St. Louis, MO, USA); Dulbecco’s modified Eagle’s medium (DMEM), foetal bovine serum (FBS), 0.25% trypsin-EDTA solution, phosphate buffered saline (PBS), penicillin, streptomycin, 3-(4,5-dimethylthiazol-2yl)-2,5-diphenyl tetrazolium bromide (MTT), dimethyl sulfoxide (DMSO) were obtained from Hyclone (Rockford, IL, USA). The HPLC column (Agilent HC-C18, 4.5 × 250 mm, 5 μm was purchased from Agilent Co. and Suzhou Bolinger Analytical Insturments Co., Ltd. (Suzhou, China). Kits for the measurement of lactate dehydrogenase (LDH), reactive oxygen species (ROS), thiobarbituric acid-reactive substance (TBARS), glutathione (GSH) estimation were purchased from Nanjing Jiancheng Bioengineering Institute (Nanjing, Jiangsu, China).

### 3.2. Preparation of Total Alkaloids of Nelumbinis Plumula (NPA)

*Nelumbinis Plumula* were dried by oven at 50 °C, and then ground to a fine powder. The peel powder (1,000 g) was impregnated for 12 h and extracted with 80% ethanol (the ratio of solid and liquid was 1:10) three times, 1 h each time. After cooling to room temperature, the extract was centrifuged at 1,500 g for 15 min and the supernatant was collected. Then it was evaporated under reduced pressure to obtain the ethanol extract, with the yield being 4.95% of the dry weight of *Nelumbinis Plumula*. Next, 5% HCL solution were used to immerse the extract until the pH was adjusted to 3. The residues were extracted three times with petroleum ether to remove the lipid contents. Then NaOH was added to the filtrate to adjust the pH to 9–10 and the mixture left standing for 24 h. The crude sample was then extracted with chloroform using liquid-liquid partition. The collected chloroform soluble fractions were evaporated to dryness under reduced pressure and called *Nelumbinis Plumula* total alkaloids (NPA), with the extraction yield being 25.52% of the dry weight of ethanol extract.

### 3.3. Determination of Total Alkaloid Content (TAT)

The TAT in samples was determined according to the acid dye complexing colorimetric method described by Elmasry [34] with some modifications. In short, the sample solution (1 mL) was mixed with acetic acid and sodium acetate buffer (5 mL, pH 6.0), followed by mixing with bromocresol green solution and chloroform. The mixture was shaken, standing and separated, and the absorbance of the chloroform layer was detected at a wavelength of 415 nm. The calibration curve was then obtained using liensinine perchlorate as standard control. The TAT was expressed by comparing with the calibration curve and all tests were performed in triplicate.

### 3.4. UV–Vis Spectrophotometric and High-Performance Liquid Chromatography (HPLC) Analysis of Alkaloid Compounds in NPA

*Nelumbinis Plumula* total alkaloid (1 mg) was dissolved in 100% methanol (analytic grade, 10 mL). The absorbance of the sample solution was scanned within the wavelength range 200–400 nm, using a model UV-1800 spectrophotometer (Shimadzu, kyoto, Japan). *Nelumbinis Plumula* alkaloid was analyzed on a HPLC (Agilent 1200) using a reversed phase Agilent HC-C18 column (4.5 × 250 mm, 5 μm) with a diode array UV-detector operating at 280 nm. A solvent system consisting of acetonitrile and 0.1% triethylamine water solution (60:40) was used as mobile phase at a flow rate of 1 mL/min. Standards such as liensine, isoliensinine, neferine were used for identification of alkaloid compounds. The identification of the alkaloid compounds was based on the retention times of their peaks and comparison with the standard compounds using the in-line diode array detector (DAD) monitoring the UV-vis absorption at 282 nm. Quantification was carried out by the external standard method. Pure standards of liensine, isoliensinine and neferine, at five different concentrations in methanol, were injected into the HPLC system, and the peak areas were calculated.

### 3.5. Determination of DPPH Radical-Scavenging Activity

Antioxidant activities of *Nelumbinis Plumula* total alkaloid was studied by DPPH method at 516 nm using a standard procedure [35]. Briefly, an aliquot of DPPH solution (4.0 mL, 0.05 mM), dissolved in ethanol, was mixed with various concentrations of samples (0.2 mL), then the mixture was kept at room temperature in the dark for 30 min. The absorbance of the resulting solution was measured at 516 nm. l-Ascorbic acid and distilled water instead of samples were used as positive and negative control, respectively. The percentage of inhibition compared with negative control group was calculated and nd the scavenging activity of the samples was expressed as 50% effective concentration (EC_50_) which represented the concentration of sample having a 50% of DPPH radical-scavenging effect [36]. All tests were performed in triplicate.

### 3.6. Cell Culture

HepG2 cells, obtained from Shanghai Center for Life Science Research Institute of Chinese Academy of Sciences cell resources, were maintained in 100 mL polystyrene culture flasks (BD-Falcon, Franklin Lakes, New Jersey, USA) with DMEM medium containing 10% foetal bovine serum (FBS), penicillin (100 units/mL), and streptomycin (100 μg/mL) under an atmosphere of 5% CO_2_ at 37 °C. HepG2 were split 1:5 every 5 days. Medium was changed every other day.

### 3.7. Cell Viability Assay

HepG2 cells were plated in 24-multi-well culture plates at 1 × 10^5^ cells per well with DMEM medium containing 10% FBS, penicillin (100 units/mL), streptomycin (100 μg/mL) and maintained in humidified 5% CO_2_ (95% air) at 37 °C. After attachment, the cells were washed twice with PBS and incubated with NPA or its compound standards in the range of 0–50 μg/mL or quercetin (25 μg/mL) as positive control for 24 h, followed by treatment with 0.55 mM *t*-BHP for 4 h. The medium was then removed and cell viability was determined by colorimetric measurement of the reduction product of (3-(4,5-dmethylthiazol-2-yl)-2,5-diphenyltetrazolium bromide (MTT) for 4 h. The medium was removed by aspiration, after which DMSO (200 μL) was added to each well to dissolve the crystals. Of formazan. Finally, using a multiplate reader, the optical density at a wavelength of 540 nm was measured, which was compared with control group to get the cell viability of each group. Triplicate samples were run for each set and averaged.

### 3.8. Measurement of LDH Leakage

HepG2 cells (1 × 10^5^ cells/mL) were treated as described above in 96-well plate with neferine at concentration of 6.25, 12.5, 25 μg/mL and 0.55 mM *t*-BHP. At the end of the incubation, 50 μL of media from each well was transferred to a new plate and measured for LDH content, According to the test kit, LDH reagent (50 μL) was added to the media and the mixture was incubated at room temperature for 30 min. The absorbance was read at 490 nm using an ELISA reader (Thermo Electron Corporation, Marietta, OH, USA). The extent of LDH leakage is expressed as U/L of the sample.

### 3.9. Induction of Intracellular ROS Production in HepG2 Cells

The intracellular ROS production was measured using dichlorofluorescin diacetate (DCFH-DA) by the method of Halliwell [37] with slight modifications. HepG2 cells were cultured in 24-multiwell culture plates at 1 × 10^5^ cells and pretreated with neferine at concentration of 12.5, 25 and 50 μg/mL or quercetin (25 μg/mL) as positive control for 24 h, followed by treatment with *t*-BHP for 4 h. Meanwhile, the fluorescent probe DCFH-DA was used to monitor the intracellular generation of ROS. After incubation, the medium was replaced with 50 μM DCFH-DA for 30 min of treatment. Then the medium was removed. Following a wash using PBS (twice), lysis buffer (cold PBS containing 1% *v/v* Triton X, 500 μM phenylmethylsulfonyl fluoride and 1% *v/v* protease inhibitor cocktail; 400 μL/well) was added to each well. The plates were left on ice for 30 min and cell lysates were collected in labelled Eppendorf tubes and centrifuged (12,000 rpm for 10 min). The supernatants were then kept at −80 °C for detection of intracellular ROS. The level of intracellular ROS was monitored using a fluorescence microscope (Nikon, Eclipse TE 2000-U, Tokyo, Japan) and a fluorometer at an excitation of 485 nm and an emission of 530 nm. Fluorescence intensity compared with the *t*-BHP alone treated group was calculated as the percentage of ROS production (%).

### 3.10. Thiobarbituric Acid-Reactive Substance (TBARS) Assay in HepG2 Cells

Malondialdehyde (MDA) was determined by the method of Ohkawa *et al*. with a slight modification [38]. In brief, after incubation of the cells described above, the medium (1 mL) was mixed with 7.5% (w/v) cold trichloroacetic acid (TCA, 1 mL) to precipitate proteins and then centrifuged at 12,000 rpm for 10 min. The supernatant was mixed with 8.1% sodium dodecyl sulfate (SDS), and distilled water and allowed to sit at room temperature for 5 min, then it was mixed with 20% acetic acid and 0.5% (w/v) thiobarbituric acid (TBA, 1 mL), and heated at 95 °C for 60 min. Finally, the mixture was centrifuged at 12,000 rpm for 10 min. Lipid peroxidation products were estimated by measuring the concentration of thiobarbituric acid reaction substances (TBARS) by measuring fluorescence at 530 nm excitation/552 nm emission. Triplicate samples were run for each set and averaged.

### 3.11. Glutathione Estimation

Intracellular GSH contents were estimated using a GSH/GSSG recycling method with some modification [39]. Cell lysates were obtained as above and a lysates sample (50 μL) was transferred for assay in another 96-well plate. For GSH, in brief, 10% trichloroacetic acid was added to the sample. After centrifugation (5000 × g, 10 min), o-phthalyldialdehyde (OPT) was added to the supernatant and incubated for 40 min at room temperature. Fluorescence at 425 nm was determined with the excitation at 350 nm. Fluorescence intensity compared with control group was calculated as the percentage of GSH production (%).

### 3.12. Statistical Analysis

All studies were conducted with three independent experiments, and values were expressed as means ± standard deviation (SD). The data were statistically analyzed by one-way ANOVA, using Tukey’s post-hoc test and applying a significance level of *p* ≤ 0.05.

## 4. Conclusions

In summary, we examined the effect of total alkaloid and alkaloid compounds from *Nelumbinis Plumula* on cytotoxicity in HepG2 cells with oxidative stress induced by *t*-BHP. Total alkaloid part showed significant improvement effects and the major components in this part were tentatively identified to be the alkaloids liensinine, isoliensinine and neferine by HPLC analysis, with the contents being 2.57%, 4.38% and 9.52% of the dry weight of NPA. Among them, the neferine component showed the strongest activities. The protective effect against oxidative stress induced by *t*-BHP may be associated with decreaes in ROS formation, TBARS generation, LDH release and increases in GSH levels in a concentration dependent manner, suggesting their involvement in the cytoprotective effects against oxidative stress.
